# Supplementary material for: Patterns of Intron Gain and Loss in Fungi
Source: PLoS Biol. 2004 Nov 30;2(12):e422. doi: 10.1371/journal.pbio.0020422 (PMC532390; doi:10.1371/journal.pbio.0020422)
Supplement: Table S1 — Also available at http://genes.mit.edu/NielsenEtAl/. (4.3 MB ZIP). [file pbio.0020422.st001.zip › NielsenEtAl/html/505.html]

AN0874.1.NCU08983.1.MG06313.1.FG05389.1


```
 CLUSTAL W (1.82) Multiple Sequence Alignments - Introns Inserted


Sequence 1: MG06313.1	484 aa
Sequence 2: FG05389.1	624 aa
Sequence 3: NCU08983.1	625 aa
Sequence 4: AN0874.1	601 aa
Alignment Length: 654 aa
Number Identitical Residues: 177 aa
Alignment Score (without introns) 10171


MG06313.1 	----------------------------------------------------~-------
NCU08983.1	---MSNPEQTLGVTPPISTTLPTDAEKRQTEALLKELRAQGTFESQAETDKR~WAVLADL
FG05389.1 	----MASDQAFGVTPPISVQLPTEAELRQSDALLEELKRQKTFESPAETAKR2EEVLASI
AN0874.1  	MAAAPSTTRQWGVTPPISTTLPTAQELSANDDLIAELKLQNNFESPAETERR2KQVLQLL
          	 ::   .    . :.. :   .:  .   ..    .   . . .: :.:           

MG06313.1 	--------------------------------------------~---------------
NCU08983.1	QRITDEFVKRAAQEKEPHNAILIRDARGRIFTYGSFRLGVYGPG~SDIDTLVVVPKYVTV
FG05389.1 	QIICDAFVRRVAEEKEPKNEVLVRNARGRVFTYGSYRLGVYGPG~SDIDTLIVAPRYVTR
AN0874.1  	QRVTIEFVKVVSRKKG-LSQAAVEAAGGKIFTYGSYRLGVYGPG1SDIDTLVLGPKHVVI
          	.          : ..   .      : .   : .:   .  ... :. .:    .     

MG06313.1 	-------------MAPPNAITDLSVVVDAFVPIIKFEFSGISIDLIFSRIATMKQLPADP
NCU08983.1	KQYFDIFPNLLVEMAPPGAITDLTPVPEAFVPIIKFEYSGISIDLIFCSIQSLRQLPDDK
FG05389.1 	EDYFKYFPDLLVSMAPKDAITDMAVVTDAFVPIIKFEYFGISIDLIFSRIIQKQLAPDFK
AN0874.1  	DDFFAEFPPLLESMAPQGAVEKITPVPDAFVPLIKLELSGISIDLIFARLIVPSIP---L
          	..     .    .*** .*: .:: * :****:**:*  ********. :          

MG06313.1 	NWNLSDSNLLRGLDEKELRSVNGTRVTDEIISLVPEQNVFRTALRAIKLWAQR~RAIYGN
NCU08983.1	DWNLNSNNLLRGLSENEVRSLNGTRVTDEILALVPEEKTFKLALRAIKLWAQR~RAIYAN
FG05389.1 	--DLKDSGLLRGLDEAELRSLNGTRVTDEILTLVPEQSTFKNALRAIKLWAQR~RAVYAN
AN0874.1  	NLDLKNNDYLRGLDEKEVRSLNGTRVTDEILELVPQQKTFRLALRAIKLWAQR1RAIYSN
          	. :*.... ****.* *:**:*********: ***::..*: *********** **:*.*

MG06313.1 	IVGFPGGVAWAMLVARVCQLYPRATASVVVNKFFCILMKWPWPQPVLLKAIEDGPLQARI
NCU08983.1	IMGYPGGVAWAMLVARVCQLYPKATSAVIVRKFFTIMLNWPWPLPVLLKNIEYNQSIT--
FG05389.1 	IMGFPGGVAWAMLVARVCQLYPKATPAVIVNKFFLVISQWRWPQPVLLKPIEGGPLPVRV
AN0874.1  	IVGFPGGVAWAMLVARVCQLYPQATGSVIVGKFFRIMNKWAWPQPVLLKPIEDGPLQMKV
          	*:*:******************:** :*:* *** :: :* ** ***** ** .      

MG06313.1 	WNPK0IYPGDRYHLMPIITPAYPSMCATFNITKSSKDVIGRELVKAADLTNLIMVGKLPW
NCU08983.1	---R0IYPSDRNHKMPIITPSYPSMCATHNVGRSSMAVIQQELERGVQISEEIMLGKRPW
FG05389.1 	WNPK0LYKGDTFHLMPVITPAYPSMCATFNITRSSMTIINRELKRALQISESIMVGMRPW
AN0874.1  	WNPK0IYHGDRFHLMPIITPAYPSMCATHNISMSTKAVILRELQRGGDIVDKIWAKQATW
          	 ..: :* .*  * **:***:*******.*:  *:  :* :** :. :: : *     .*

MG06313.1 	KDLFQKHTFFTKDFKYYIQVIAASKDKESHKLWSGFVESKVRLLVQGLERHESIAVARPF
NCU08983.1	KDLFEKHTFFTNGFKFYLTVISSGRTKEAQNTWSGFVESRVRVLVQKLELHPSIALARPF
FG05389.1 	SDLFAKHSFFTAGYKYYISVISAAKTKESHTIWSGYVESKVRMLVQKLEQHQSIALAHPF
AN0874.1  	NDLFKKHSFFTNDYKYYLGVTASSRTKEAESVWSGLVESKVRHLVGALDRKPTIAVAHPF
          	.*** **:*** .:*:*: * ::.: **:.. *** ***:** **  *: : :**:*:**

MG06313.1 	IKGYDRSHRYYDDSQFNQILEGSLANLCQDKDPQPE------AKPEVP--VKEEPVSNGT
NCU08983.1	NKGYDRVHRCKTDAQVEEIQTGNLTYMVKPTDIAANGSAKNDSKVEVKAEIKTEDGVARE
FG05389.1 	NKGYERQHRCQNEKEIEAVQEGSLDFLSKEDATEPTTIKIEPAVAELP--IKTEDGEDGK
AN0874.1  	PKGFERIHIVSNEEEAEAVKNGSLKYQDKGTKTETT-----------------DEIKDAA
          	 **::* *    : : : :  *.*    :     .                  :      

MG06313.1 	HTNGIAVKDE------VAIK---QEGHARIKSEDCVDPQPLTENIPGFKGPSS---DMYT
NCU08983.1	PTVPAVKEEPNDVVHLGTVPLKPEDNSVKPEANDYDEDKVKLEDIPEKEPELE----IYT
FG05389.1 	SIKAENGENG------LTVKSKTTEDNGPITAEPFN-PETSSDTKPSETPAVSGAINVYT
AN0874.1  	HQAAAQSGRE--------------DTDVPGAANNTAANDGR---------------IIYT
          	                        :      ::     .                  :**

MG06313.1 	TTFYIGLELRP-~---~-----D1LSFQVNDFKWLCYDWAKYKEDLTELCFVNIKDLRS2
NCU08983.1	TNHYIGLQLVEG~A--~--KSLD~LSREVDDFKAMCTSNDIFKAELMGLT---IQHVRN~
FG05389.1 	TTHYIGLELAAG1A--~--KSLD~LSYQVEEFKELCTSWQKYKDDLKDVVSIGIQHVRN2
AN0874.1  	TTYYIGLELKPV~EPG1ASRSLD~ISSDAQFFKSMCTSWPGYQPGINDLS---ITHVRN2
          	*..****:*     .. :: : * :* :.: ** :* .   ::  :  :    * .:*. 

MG06313.1 	FALPDDIFGPGETKPV---KRLVKKKVPVDRKRPAN---~E0DNQAPAKRHQPATVGSGV
NCU08983.1	FDLPDDVFEPGETKPV---RPVKKK----KRMAEGE---~H~ANGPMAKRQQMATAPPTI
FG05389.1 	FNLPDDVFEDGEKKPQ---KKSAAKGAANNKKRGPP---~E0ETPPPAKRQQSSIAAAG-
AN0874.1  	YDLPEDLFQEGEARPTRPKKKVVKKVPAGGQKRSFDSVD0D~PSNPAAKRQVTDSYATAT
          	: **:*:*  ** :*  ..:    *  .  :     : . .  . . ***:      .  

MG06313.1 	----
NCU08983.1	KSEH
FG05389.1 	----
AN0874.1  	AQPA
          	 .
```
